# Adhesion of *Salmonella* to Pancreatic Secretory Granule Membrane Major Glycoprotein GP2 of Human and Porcine Origin Depends on FimH Sequence Variation

**DOI:** 10.3389/fmicb.2018.01905

**Published:** 2018-08-22

**Authors:** Rafał Kolenda, Michał Burdukiewicz, Juliane Schiebel, Stefan Rödiger, Lysann Sauer, Istvan Szabo, Aleksandra Orłowska, Jörg Weinreich, Jörg Nitschke, Alexander Böhm, Ulrike Gerber, Dirk Roggenbuck, Peter Schierack

**Affiliations:** ^1^Institute of Biotechnology, Faculty Environment and Natural Sciences, Brandenburg University of Technology Cottbus-Senftenberg, Senftenberg, Germany; ^2^Department of Biochemistry and Molecular Biology, Wrocław University of Environmental and Life Sciences, Wrocław, Poland; ^3^Faculty of Mathematics and Information Science, Warsaw University of Technology, Warsaw, Poland; ^4^National Salmonella Reference Laboratory, Federal Institute for Risk Assessment (BfR), Berlin, Germany; ^5^GA Generic Assays GmbH, Berlin, Germany

**Keywords:** FimH, *Salmonella*, GP2, receptor, intestine, host-specificity, Type 1 fimbriae, host-pathogen interactions

## Abstract

Bacterial host tropism is a primary determinant of the range of host organisms they can infect. *Salmonella* serotypes are differentiated into host-restricted and host-adapted specialists, and host-unrestricted generalists. In order to elucidate the underlying molecular mechanisms of host specificity in *Salmonella* infection, we investigated the role of the intestinal host cell receptor zymogen granule membrane glycoprotein 2 (GP2), which is recognized by FimH adhesin of type 1 fimbriae found in *Enterobacteriaceae*. We compared four human and two porcine GP2 isoforms. Isoforms were expressed in Sf9 cells as well as in one human (HEp-2) and one porcine (IPEC-J2) cell line. *FimH* genes of 128 *Salmonella* isolates were sequenced and the 10 identified FimH variants were compared regarding adhesion (static adhesion assay) and infection (cell line assay) using an isogenic model. We expressed and characterized two functional porcine GP2 isoforms differing in their amino acid sequence to human isoforms by approximately 25%. By comparing all isoforms in the static adhesion assay, FimH variants were assigned to high, low or no-binding phenotypes. This FimH variant-dependent binding was neither specific for one GP2 isoform nor for GP2 in general. However, cell line infection assays revealed fundamental differences: using HEp-2 cells, infection was also FimH variant-specific but mainly independent of human GP2. In contrast, this FimH variant dependency was not obvious using IPEC-J2 cells. Here, we propose an alternative GP2 adhesion/infection mechanism whereby porcine GP2 is not a receptor that determined host-specificity of *Salmonella*. *Salmonella* specialists as well as generalists demonstrated similar binding to GP2. Future studies should focus on spatial distribution of GP2 isoforms in the human and porcine intestine, especially comparing health and disease.

## Introduction

Zymogen granule membrane glycoprotein 2 (GP2), a microbiota-sensing and immune modulating molecule, contains an N-terminal signal peptide, an EGF-like domain, a zona pellucida domain, and a C-terminal glycosylphosphatidylinositol (GPI)-anchor (Jovine et al., 2005; Yu and Lowe, 2009; Werner et al., 2012). GP2 is the most abundant protein in the pancreatic secretory granule membrane (Ronzio et al., 1978). During pancreatic secretion, GP2 is cleaved from the secretory granule membrane and released into the intestine. GP2 sequence orthologs can be found among mammals, but there is no information about GP2 in other classes of vertebrates. Various numbers of alternative splice variants are expressed in different animals (Fukuoka, 2000). Four isoforms have been predicted for humans, two in cattle and pig and one in mouse (Maglott et al., 2005).

The role of GP2 in the pancreas is poorly elucidated. The proposed role in sorting of proteins to the zymogen granule or regulation of granule membrane recycling (Fritz et al., 2002) could not be confirmed by a murine GP2 knock-out model (Yu et al., 2004). Of note, GP2 was identified as intestinal M cell marker in mice and humans (Hase et al., 2009). Further, GP2 was found on cells in various mucous glands in the murine digestive, respiratory, and genital tracts (Kimura et al., 2015, 2016), on human cells vital to mucosal innate and adaptive immune responses and on enterocytes (Roggenbuck et al., 2009; Werner et al., 2012).

*Salmonella* uses M cells and enterocytes as major gateways for entry in the mucosal epithelium (Sansonetti, 2004). Interestingly, they also trigger the conversion of enterocyte cells into M cells (Tahoun et al., 2012). M cells are epithelial cells and specialized in the transport of luminal antigens and bacteria across the gut (Williams and Owen, 2015). Translocation of *Salmonella* through M cells is Type Three Secretion System-1 and -2 independent (Martinez-Argudo and Jepson, 2008). Thus, adhesion thereof should be possible by other factors. However, receptors on the cell surface remained largely unknown (Baumler et al., 1996). Studies have shown that type 1 fimbriae (T1F), expressed by *Salmonella* and *Escherichia coli* among others, bind to native GP2 on the apical pole of M cells, and to recombinant GP2 *in vitro* (Hase et al., 2009). Further, GP2 was confirmed as receptor for other T1F-expressing bacteria (Schierack et al., 2015).

*Salmonella* serotypes can be divided by host range and clinical signs into host-restricted and host-adapted specialists and host-unrestricted generalists (Stevens et al., 2009). Host-restricted serotypes like *S*. Gallinarum cause systemic infection in one host (called fowl typhoid/pullorum disease) and do not cause disease in any other host (Shivaprasad, 2000). Host-adapted specialists, e.g., *S*. Choleraesuis and *S*. Dublin cause mainly systemic disease in pigs and cattle, respectively, but can sporadically cause asymptomatic infections in other hosts (Nielsen, 2013). Most serovars belong to the host-unrestricted group. They are able to infect multiple hosts, resulting in gastroenteritis (Majowicz et al., 2010). T1F is one of the most common adhesins in the family of *Enterobacteriaceae* and important also in *Salmonella* pathogenicity (Yue et al., 2012). The FimH protein is located on top of the T1F shaft and directly interacts with glycoprotein-receptors (Kisiela et al., 2013). Several studies have shown that serotype-associated FimH variants of *Salmonella* specialists and generalists can differ significantly in receptor recognition or tropism to different tissue types. This can lead to changes in the course of infection (Grzymajlo et al., 2010, 2013).

So far, comparative functional studies on the interaction of human or mouse GP2 and bacteria have used only one isoform for each organism. Moreover, there has been no study comparing different isoforms of the human GP2 protein with those of other host species. We showed that binding of *E. coli* to one human GP2 isoform correlated with FimH amino acid sequences (Schierack et al., 2015). We hypothesized that also *Salmonella* pathotypes with their host-specificity and with their conserved FimH sequences would differentially bind to GP2 isoforms. Thus, we (1) recombinantly expressed two pig GP2 isoforms on the basis of tissue mRNA, (2) expressed four human and two pig GP2 isoforms in epithelial cells, (3) characterized 128 *Salmonella* isolates for FimH sequences, and (4) analyzed a possible FimH variant – GP2 isoform-specific binding using an isogenic *Salmonella* model including all 10 defined FimH variants. For extensive automated screening, we developed a novel software module for Fluorescent *in situ* Hybridization (FISH) for use with an already established automated fluorescence microscopy-based VideoScan technology. In general, the VideoScan technology enables automated imaging and analysis of fluorescent objects like microbeads or bacteria (Rödiger et al., 2013).

## Materials and Methods

### Strains

*Salmonella* isolates were obtained from the National *Salmonella* Reference Laboratory, Federal Institute for Risk Assessment (BfR) in Berlin, Germany and from Mydlak/Thorasch Diagnostic Laboratory in Cottbus, Germany. For cloning of *FimH* gene alleles, *E. coli* XL1Blue was used. For expression of porcine GP2, *E. coli* DH10Bac was used to generate bacmid DNA with subsequent expression of proteins in SF9 cells. The list of the strains is provided in **Supplementary Table 1**.

### Cloning and Expression of Porcine GP2 Isoforms

Pancreas was obtained from a freshly killed pig in the slaughterhouse. Small cuts were preserved in RNAlater solution (Qiagen). RNA was isolated with use of the RNeasy Mini Kit (Qiagen) according to the manufacturer’s protocol. The reverse transcription (RT) reaction was performed with Maxima First Strand Synthesis Kit for RT-qPCR (Thermo Scientific). Next, GP2 isoforms were cloned into pJET1.2 plasmids (Thermo Scientific) according to the manufacturer’s protocol and Sanger sequenced. In order to express the secretory form of GP2 in SF9 cells, GPI modification site prediction was done using PredGPI (Pierleoni et al., 2008) and FragAnchor (Poisson et al., 2007) software and this part of the GP2 sequence was not cloned. Porcine GP2 isoforms were cloned with the Gateway system using pDONR221 and pDEST8 plasmids (Invitrogen). Primer sequences used in this section are shown in **Supplementary Table 2**. Next, recombinant bacmids in DH10Bac bacteria were generated and isolated. Recombinant bacmid DNA was transfected into SF9 cells, and baculovirus was amplified two times in SF9 cells and titrated with BacPAK Baculovirus Rapid Titer Kit (Clontech). In the next step, recombinant GP2 was expressed in a larger volume (200 ml) in suspension culture of SF9 cells. Proteins were purified by His-tag affinity chromatography and protein purity was assessed by silver staining and Western blotting with anti-His-tag antibody. Purified porcine GP2 isoforms 1 and 2 (500 μg of each) were send to Pineda Antibody Service for rabbit immunization, antibody raising and antibody purification. Rabbits were re-immunized on days 20, 30, 40, and 61 after first immunization. After 90 days rabbits were bleed out and the total IgG fraction was purified from sera by protein A/G chromatography.

### Porcine GP2 Deglycosylation

Protein deglycosylation in denaturing and non-denaturing conditions was performed with protein deglycosylation mix (NEB). Negative control for protein digestion contained everything but enzyme mix and was incubated under identical conditions. After digestion, proteins (2 μg for each aliquot) were resolved on SDS–PAGE and stained with Coomassie brilliant blue. For blotting 200 ng of each protein was resolved on SDS–PAGE, transferred to a PVDF membrane and checked with an anti-6-His antibody and Concanavalin A (ConA).

### Sequencing, Sequence Alignment, and Comparison for *FimH* Genes of *Salmonella*

Genomic DNA was isolated from 128 *Salmonella* isolates with DNeasy Blood and Tissue Kit (Qiagen). *FimH* genes were amplified by PCR using the following reaction mix: 1 U of Phusion High-Fidelity DNA Polymerase (Thermo Scientific), Phusion HF buffer (Thermo Scientific), MgCl_2_ (at final concentration of 1.5 mM), DNA (10–50 ng), 0.5 μM of each primer and 0.2 mM dNTPs mix and water to final volume of 50 μl. PCR was started with initial denaturation (98°C, 30 s), followed by 40 cycles of denaturation (98°C, 5 s), annealing (53°C, 15 s), elongation (72°C, 45 s), and final elongation (72°C, 10 min). PCR product quality was checked on a 1% agarose gel. Subsequently, 100 μl of each PCR reaction solution was Sanger-sequenced by LGC Genomics GmbH (Berlin). Primer sequences used for amplification and sequencing are shown in **Supplementary Table 2**. Sequence reads were assembled into contigs with a CAP3 software implemented into the UGENE platform (Okonechnikov et al., 2012). Sequences were aligned and compared using ClustalX 2.1 (Larkin et al., 2007).

### Generation of *Salmonella* Isogenic Strains

A *FimH* gene deletion mutant in *Salmonella* strain Tym5744 was generated according to Datsenko and Wanner (2000) with slight modifications. As Tym5744 was ampicillin resistant, we used pKD46-Gm and pCP20-Gm instead of original pKD46 and pCP20 plasmids (Doublet et al., 2008). The mutant was named Tym5744Δ*fimH*. All 10 *fimH* alleles found in 128 analyzed *Salmonella* isolates were cloned into a pACYC177 plasmid. Three plasmids (three variants) were already included in another study (Grzymajlo et al., 2017). Next, plasmids containing *fimH* variants were transformed into Tym5744Δ*fimH*. Primer sequences used in this section are shown in **Supplementary Table 2**. We carried out an extensive screening of 128 *Salmonella* isolates for their expression of T1F and binding propensities to anti-FimH antibodies, using a static adhesion assay (see the Section “Static Adhesion Assay”). Strain Tym5744 showed robust expression of T1F and binding to anti-FimH antibodies and thus a proper T1F expression machinery and was therefore used for further analysis.

Expression of FimH in FimH variants was quantified by the following protocol. Bacteria were grown in 1.5 ml Eppendorf tubes under static conditions for 48 h at 37°C in LB medium supplemented with 50 μg/mL kanamycin. The following steps were performed in 1.5 ml Eppendorf tubes. Bacteria were washed once by centrifugation for 5 min at 4000 × *g* with PBS. Bacterial cells were fixated in PBS-PFA (4%) solution for 1 h at 4°C. Subsequently, bacteria were washed three times with PBS and incubated with BSA (= blocking solution, 1% BSA in PBS) for blocking for 30 min at room temperature. Bacteria were washed once with blocking solution. Bacteria were incubated with the anti-FimH antibody (dilution 1:200) in blocking solution for 1 h at room temperature. Bacteria were washed with the blocking solution. Bacteria were incubated with the secondary antibody Alexa Fluor 647 Anti-Rabbit IgG (1:250, Dianova) for 1 h at room temperature in the dark. Bacteria were washed three times with PBS. The OD_600_ was measured. Now bacterial suspensions in PBS were pipetted into wells of a 96-well plate. Fluorescence of the well was measured using VideoScan. Fluorescence intensity was the parameter for FimH expression. As negative control, the FimH deletion mutant was included. Background fluorescence of the FimH mutant was subtracted from FimH fluorescence of FimH variants.

### Static Adhesion Assay

For static adhesion assays, proteins were coated on 96-well plates. Proteins were diluted in coating buffer (0.1 M Na_2_CO_3_, pH = 9.6), 50 μl of solution was pipetted to each well of a 96-well plate (Nunc MaxiSorp, flat-bottom) and incubated overnight in 4°C. On the next day, plates were washed once with PBS/BSA 1% and dried at room temperature for 30 min. The Anti-FimH antibody was coated at a dilution of 1:250, GP2 isoforms at concentrations of 2.5 μg/ml, horseradish peroxidase (HRP), and RNase B at concentrations of 5 μg/ml (Grzymajlo et al., 2010). In total, the following proteins were included: four human GP2 isoforms expressed in SF9 cells (GA Generic Assays), two porcine GP2 isoforms expressed in SF9 cells (this study), HRP purified from horseradish (Sigma, Cat No. P6278), RNase B isolated from bovine pancreas (Sigma, Cat No. R7884) and rabbit polyclonal anti-FimH antibody (Wrocław University of Environmental and Life Sciences, Poland). *Salmonella* isogenic strains were grown in 1.5 ml Eppendorf tubes filled with 500 μl of LB broth with kanamycin (50 μg/ml) under static conditions at 37°C to induce T1F expression (Old and Duguid, 1970). After 48 h bacteria were diluted to 5 × 10^7^/ml in PBS/BSA 1% and 100 μl of bacteria were applied to each well of 96-well plates coated with proteins. Bacteria were incubated for 2 h at room temperature. Plates were washed three times with PBS. Attached bacteria were fixed with 50 μl of 4% PFA in PBS for 30 min at 4°C. Plates were washed three times with PBS. Attached bacteria were stained with 50 μl of propidium iodide (10 μg/ml in ddH_2_O) for 15 min at room temperature. Plates were washed three times with PBS. DAPI beads (PolyAn) served as positive control for our VideoScan analysis and 75 μl of a DAPI bead solution was pipetted into each well (Schierack et al., 2015). At least three independent experiments with a minimum of three replicates were performed. For each well, 40 images were taken per well and the bacteria were counted using our VideoScan technology. The median number of bacteria was calculated for these 40 images. This was followed by calculating the median value of all respective wells for replicate experiments. Finally, the median value was determined for all experiments.

### Generation of GP2-Expressing Cell Lines

GP2-expressing cell lines were generated with the Lenti-X Lentiviral Expression System (Clontech). First, GP2 isoforms were cloned into a pLVX-IRES-puro plasmid. Next, puromycin resistance of cell lines was tested by measuring cell viability and proliferation with the MTT assay (Owczarek et al., 2013). Lentiviruses were generated according to the manufacturer’s protocol. HEp-2 cells were transduced with lentiviruses containing human GP2 isoforms and IPEC-J2 cells (Schierack et al., 2006) were transduced with lentiviruses containing porcine GP2 isoforms. Both cell lines were transduced with empty lentivirus as negative controls (empty lentivirus control). Confirmation of GP2 expression in transduced cell lines was done with RT-PCR. RNA from transduced cells was isolated with the RNeasy Mini Kit (Qiagen), according to the manufacturer’s protocol. Next, the reverse transcriptase reaction was performed as in the Section “Sequencing, Sequence Alignment, and Comparison for *FimH* Genes of *Salmonella*.” PCR was performed using standard conditions and primers. RPLP0 was used as a reference gene (George et al., 2016). Primer sequences used in this section for cloning and RT PCR are shown in **Supplementary Table 2**. To confirm GP2 expression at the protein level, indirect immunofluorescence (IIF) was used following a standard protocol. Cell lines were tested for GP2 expression with rabbit polyclonal antibodies raised against human GP2 isoform 1 (GP2Ho#1; HEp-2) or porcine GP2 isoforms 1 and 2 (GP2Su#1 and GP2Su#2; IPEC-J2). Western Blot was performed with antibodies GP2Ho#1 and GP2Su#2. Flow cytometry was done with standard protocols using the same antibodies like for IIF.

### Cell Line Adhesion/Infection Assays

Cell lines were seeded in 96-well plates and assays were performed when cells reached confluency. Starting an assay, cell lines were washed with PBS and D-MEM/Ham’s F12 medium (Millipore) supplemented with 5% bovine serum (Millipore) and 2 mM L-glutamine was added. *Salmonella* isogenic strains were grown as in the Section “Static Adhesion Assay.” After OD_600_ measurement, 5 × 10^6^ bacteria in 50 μl were pipetted into each well. For mannose blocking assays, bacteria were first pre-incubated for 30 min with 0.2 M mannose. Bacteria were incubated for 2 h on cells. Plates were washed three times with PBS. Cells and bound bacteria were fixed with 50 μl of 4% PFA in PBS for 1 h at 4°C. Plates were washed three times with ddH_2_O. Cells were dehydrated with 50 μl of 95% ethanol for 5 min, dried and stored at 4°C until fluorescence *in situ* hybridization (FISH). For FISH staining hybridization buffer (0.9 M NaCl, 20 mM Tris–HCl, 0.01% SDS, 15% formamide) and washing buffer (0.9 M NaCl, 20 mM Tris–HCl, 0.01% SDS) were freshly prepared and pre-warmed to 46°C. FISH probe EUB338 Atto647N was diluted in hybridization buffer to a final concentration of 5 ng/μl and 40 μl was pipetted into each well. Plates were incubated in a humid chamber for 1 h at 46°C and washed once with washing buffer. Plates were incubated for 10 min at 48°C with washing buffer. Nuclei were stained with DAPI (50 μg/ml in ddH_2_O) and washed once with ddH_2_O. Plates were dried at room temperature for VideoScan analysis. At least three independent experiments with a minimum of three replicates were performed.

### VideoScan

VideoScan is a fluorescence microscopy-based technology (Rödiger et al., 2013) which automatically takes images from wells of cell culture plates and analyses fluorescent objects. To use this technology in counting bacteria, we first proceed to make them fluorescent by using the FISH technology (see the Section “Cell Line Adhesion/Infection Assays”). The VideoScan module works with a 20× magnification objective and first focuses in the well on DAPI stained nuclei or DAPI beads. After focusing, the equipment takes an image of the well, and fluorescent bacteria are then counted. A total of 40 images per well were taken during read-out. Three to five independent experiments with triplicates for each FimH variant and recombinant protein/cell line were prepared and measured.

### Statistical Analysis

Statistical analysis was performed using the R software (R Development Core Team, 2013). The RStudio and RKWard v. 0.6.9z + 0.7.0 + devel1 with integrated development environments were used for the R script development. Central tendency and spread were calculated as median and median absolute deviation. Pairwise Mood’s median test from the RVAideMemoire package was used to perform pairwise comparisons between groups with a Benjamini and Hochberg’s (1995) correction to account for multiple testing. All figures were made using the ggplot2 package (Wickham, 2010).

## Results

### Cloning and Expression of Porcine GP2 Isoforms

**FIGURE 1:**
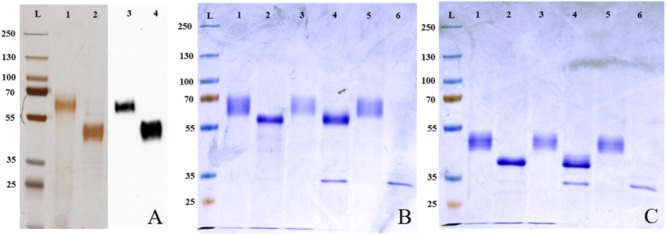
Expression and deglycosylation of porcine GP2. **(A)** Silver staining and Western blotting with an anti-6-His antibody of purified porcine GP2 isoforms. Lane L: protein marker (kDa), lanes 1 and 3: porcine GP2 isoform 1 (GP2Su#1), lanes 2 and 4: porcine GP2 isoform 2 (GP2Su#2), lanes 1 and 2: silver staining, lanes 3 and 4: Western Blot. **(B,C)** After deglycosylation of recombinant porcine GP2 isoform 1 [GP2Su#1, **(B)**] and porcine GP2 isoform 2 [GP2Su#2, **(C)**] proteins were validated by coomassie brilliant blue staining. Lane L: protein ladder (kDa), lane 1: native, non-digested protein, not incubated in 37°C; lane 2: protein digested in denaturing conditions; lane 3: protein incubated in denaturing conditions without enzymes; lane 4: protein digested in native conditions; lane 5: protein incubated in native conditions without enzymes; lane 6: enzyme mix only.

The mRNA isolation from porcine pancreatic tissue, cDNA amplification, and sequencing resulted in two GP2 sequences identical with GenBank sequences XM005662102 and XM003124571. Our two sequences were submitted to GenBank with accession numbers KU665994 and KU665995. Porcine and human GP2 isoforms had 76% sequence identity. Comparison of the two porcine and the four human isoforms are shown in **Supplementary Figure 1**. For expression in SF9 cells, the GPI anchor sequence was removed and only “secretory forms” of GP2 were cloned. Proteins were expressed and purified and purity was checked (**Figure 1A**).

### Porcine GP2 Deglycosylation

**FIGURE 2:**
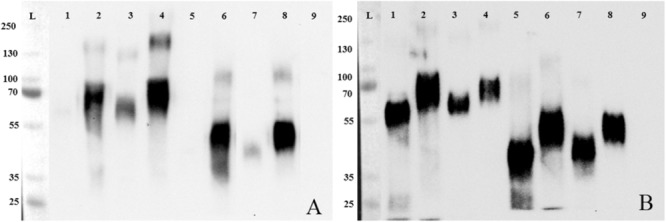
Deglycosylation of GP2, Western blotting. After deglycosylation of recombinant porcine GP2 isoform 1 (GP2Su#1) and porcine GP2 isoform 2 (GP2Su#2) proteins were validated by Western Blot using ConA **(A)** and an anti-6-His antibody **(B)**. Lane L: protein ladder (kDa), lane 1: GP2Su#1 digested in denaturing conditions; lane 2: GP2Su#1 incubated in denaturing conditions without enzymes; lane 3: GP2Su#1 digested in native conditions; lane 4: GP2Su#1 incubated in native conditions without enzymes; lane 5: GP2Su#2 digested in denaturing conditions; lane 6: GP2Su#2 incubated in denaturing conditions without enzymes; lane 7: GP2Su#2 digested in native conditions; lane 8: GP2Su#2 incubated in native conditions without enzymes; lane 9: enzyme mix only.

FimH binds to carbohydrate residues of receptor proteins. We validated glycosylation of proteins by deglycosylation which results in a shift in band height in SDS–PAGE. Glycosylation was incomplete under native and complete under denaturating conditions (**Figures 1B,C**). Results were confirmed by blotting with ConA. ConA is a lectin isolated from *Canavalia ensiformis* which recognizes branched alpha-mannosidic structures in high mannose type, hybrid type, and biantennary complex type N-glycans. ConA only bound well to non- and incomplete digested proteins (**Figures 2A,B**). In conclusion, porcine GP2 was expressed as glycosylated protein.

**FIGURE 3:**
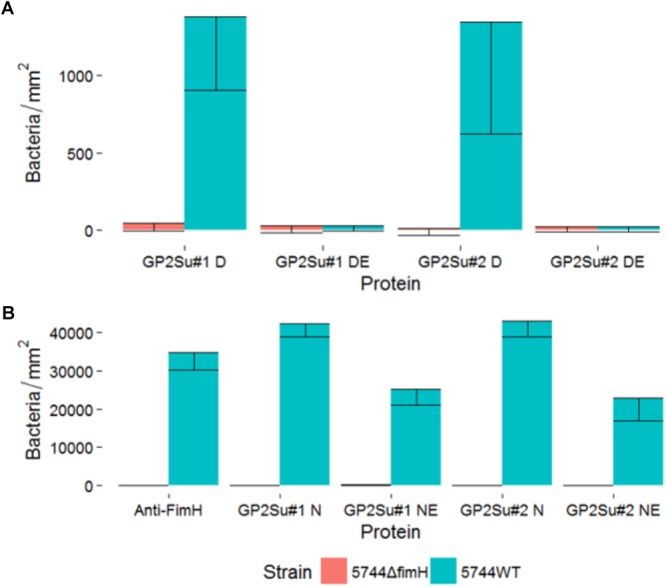
Static adhesion assay with deglycosylated GP2. *S*. Typhimurium strain 5744 wild type (5744WT, blue color) and it is *FimH* gene deletion mutant (5744ΔfimH, red color) were incubated with **(A)** GP2 deglycosylated under denaturing conditions and **(B)** GP2 partially deglycosylated under native conditions. Expression of type 1 fimbriae was confirmed by binding to an anti-FimH antibody which was included [first bar in **(B)**]. *X*-axis: D, GP2 incubated in denaturing conditions without enzymes; DE, GP2 digested in denaturing conditions; N, GP2 incubated in native conditions without enzymes; NE, GP2 digested in native conditions; GP2Su#1, porcine GP2 isoform 1; GP2Su#2, porcine GP2 isoform 2; Anti-FimH, antibody against FimH.

To confirm, that glycosylation is necessary for *Salmonella* binding, static adhesion assays with glycosylated and deglycosylated porcine GP2 were performed. Bacteria did not bind to complete deglycosylated GP2 and binding was decreased by 59% (isoform 1) and 53% (isoform 2) using GP2 incompletely deglycosylated which shows that *Salmonella* bound to carbohydrate residues (**Figures 3A,B**). Denaturation of GP2 by incubation with DTT in Glyprotein Denaturing Buffer lowered binding by destruction of disulfide bonds which stabilize protein tertiary structure.

*Enterobacteriaceae* bind to carbohydrate residues via FimH, the tip protein of T1F. To validate the role of FimH in GP2 binding, we mutated *Salmonella* Typhimurium wild type (Tym5744) which resulted in Tym5744Δ*fimH.* In contrast to Tym5744 which bound to anti-FimH antibodies, Tym5744Δ*fimH* did not which confirmed proper mutation (**Figures 3A,B**). Consistently, Tym5744 bound to GP2 but not Tym5744Δ*fimH* confirming the glycoprotein-FimH interaction (data not shown).

### Sequencing, Sequence Alignment, and Comparison for *FimH* Gene

*FimH* genes of 128 *Salmonella* isolates were sequenced. We detected 12 DNA sequence alleles and 40 variable sites. Translation resulted in 11 protein sequence variants (FimH variants) with 18 variable sites (**Table 1**). This included one new and unpublished FimH variant for *S*. Choleraesuis and one unpublished and new for *S*. Typhimurium. New variants were proofed by a second amplification and sequencing run. Among 40 *S.* Typhimurium isolates 4 *FimH* gene alleles were present which resulted in four protein variants (Tym1-4). One variant was shorter due to translation termination after 79 codons and present in only one isolate. Among 34 *S*. Enteritidis isolates two *FimH* gene alleles were present – with only one difference which was a silent mutation – which resulted in one single FimH variant (Ent). Nine *S.* Choleraesuis isolates did not possess the *FimH* gene and three *FimH* gene alleles resulted in three protein variants (Chol1–3). All 20 *S*. Dublin isolates had the identical *FimH* gene sequence and thus one protein variant (Du). In 19 *S*. Gallinarum isolates, two gene alleles and two variants were present. The first variant is typical for *S*. Gallinarum biovar Pullorum (Pull) and was present in 16 isolates. The second variant, typical for *S*. Gallinarum biovar Gallinarum (Gall), was found in three isolates.

**Table 1:** Amino acid variation in *Salmonella* FimH sequence variants.

Isolate∖Position	13	50	57	63	78	80	89	101	126	131	137	166	182	222	245	279	285	317
LT2	A	P	P	V	T	R	Q	N	L	Y	K	T	T	A	V	S	T	I
Tym1 (33)	.	.	.	.	.	.	.	.	.	.	.	.	.	.	.	.	.	.
Tym2 (1)	.	.	.	.	.	.	.	.	.	.	.	R	.	.	.	.	.	.
Tym3 (5)	.	.	.	.	.	.	.	.	.	.	.	.	.	.	A	.	.	.
Tym4 (1)	.	.	.	.	.	STOP	.	.	.	.	.	.	.	.	.	.	.	.
Ent1 (34)	.	.	.	.	.	.	.	.	R	S	M	.	.	.	.	.	.	N
Chol1 (1)	.	S	.	.	.	.	.	.	.	S	.	.	.	.	.	G	.	N
Chol2 (1)	.	.	L	G	.	.	R	.	R	S	.	.	.	.	.	.	.	N
Chol3 (4)	.	.	L	.	.	.	R	.	R	S	.	.	.	.	.	.	.	N
Du (20)	.	.	.	.	.	.	.	S	R	S	M	.	.	.	.	.	I	N
Gall (16)	T	.	.	.	I	.	.	.	R	S	M	.	.	.	.	.	.	N
Pull (3)	.	.	.	.	I	.	.	.	R	S	M	.	S	V	.	.	.	N

*FimH sequence variants of *S*. Typhimurium (Tym1–4), *S.* Enteritidis (Ent1–2), *S.* Choleraesuis (Chol1-3), *S.* Dublin (Du), *S.* Gallinarum biovar Pullorum (Pull), and *S.* Gallinarum biovar Gallinarum (Gall) discovered in this study were aligned with the FimH sequence of *S.* Typhimurium reference strain LT2 (LT2). Only polymorphic positions in the FimH protein are shown. Residues identical to the amino acid sequence of *S.* Typhimurium LT2 are indicated by dots and STOP means stop codon. Numbers in brackets stand for a number of isolates with the particular FimH variant.*

### Generation of *Salmonella* Isogenic Strains and Static Adhesion Assays

**FIGURE 4:**
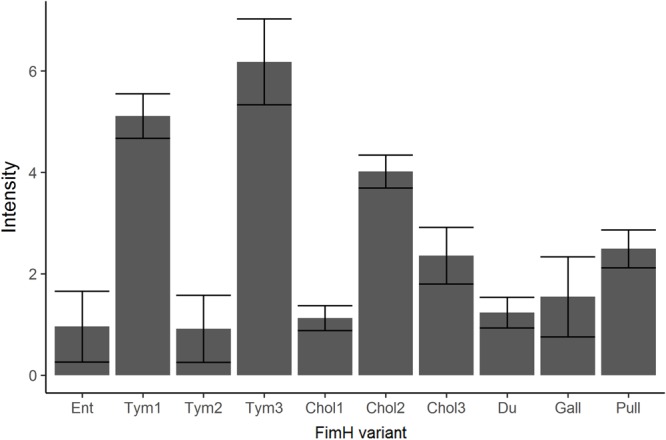
Expression of FimH in FimH variants. After complementation of Tym5744Δ*fimH* with *fimH* variants in the identical plasmid, FimH expression was tested by staining bacteria in suspension with an anti-FimH antibody. This was followed by staining with a fluorescence-labeled secondary antibody. After pipetting stained bacteria into wells of a 96-well plate, fluorescence of the well was quantified by VideoScan. Background signal of Tym5744Δ*fimH* was subtracted from signals of FimH variants. Ent: FimH variant of *S.* Enteritidis, Tym1-3: FimH variants of *S.* Typhimurium, Chol1-3: FimH variants of *S.* Choleraesuis, Du: FimH variant of *S.* Dublin, Gall: FimH variant of *S.* Gallinarum, Pull: FimH variant of *S.* Pullorum. The data are shown as median values and median absolute deviation (MAD) of three separate experiments in triplicate wells.

**FIGURE 5:**
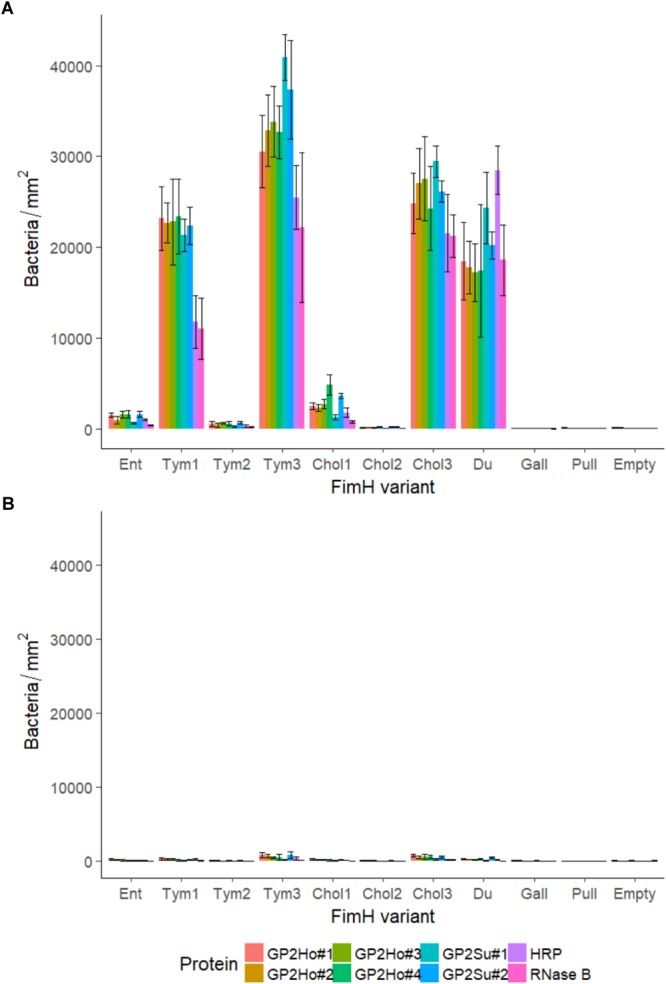
Binding of *Salmonella* to glycoproteins. FimH isogenic strains of Tym5744Δ*fimH* were incubated with **(A)** various proteins including human GP2 isoforms 1–4 (GP2Ho#1–4), porcine GP2 isoforms 1–2 (GP2Su#1–2), horseradish peroxidase (HRP) and RNase B; **(B)** identical with **(A)** but with mannose pre-incubation. If one FimH variant bound well to one GP2 isoform then this variant bound also well to other isoforms of the host species. If one FimH variant bound well to human GP2 this variant also bound well to porcine GP2. Binding to all glycoproteins was mannose-dependent **(B)**. Ent: FimH variant of *S*. Enteritidis, Tym1-3: FimH variants of *S*. Typhimurium, Chol1-3: FimH variants of *S*. Choleraesuis, Du: FimH variant of *S*. Dublin, Gall: FimH variant of *S*. Gallinarum, Pull: FimH variant of *S*. Pullorum, Empty: FimH mutant without FimH complementation. The data are shown as median values and median absolute deviation (MAD) of three separate experiments in triplicate wells.

In order to investigate the influence of *FimH* gene sequence variations on binding to GP2, we created isogenic strains. Tym5744Δ*fimH* was complemented with all FimH variants excluding the non-functional 79 amino acid short Tym variant. First we tested for FimH expression. As shown in **Figure 4**, though in an identical genetical background, FimH expression varied between FimH variants. Isogenic FimH variants were tested for binding to GP2 in a static adhesion assay. We observed three FimH binding phenotypes: the high binding phenotype included Tym1, Tym3, Chol3, and Du, the low binding phenotype Ent, Tym2, and Chol1 and the no-binding phenotype Chol2, Gall, and Pull. There was no difference in binding to the two porcine GP2 isoforms, since a variant that bound readily to isoform 1 also did so to isoform 2 (**Figure 5A**). Since FimH expression between the FimH variants was different, we have normalized the data for static adhesion assay (binding to glycoproteins) to FimH expression for each variant. This normalization, however, does not change the experimental outcome compared to non-normalized data (**Supplementary Figure 2**), since we still observe three FimH binding phenotypes: the high binding phenotype included Tym1, Tym3, Chol3, and Du; the low binding phenotype Ent, Tym2, and Chol1; and the no-binding phenotype Chol2, Gall, and Pull. Binding of FimH variants to GP2 thus depends on FimH sequences as well as FimH expression.

We hypothesized that GP2 as host cell receptor selects for host-restricted or host-adapted *Salmonella* serotypes. We compared adhesion to porcine GP2 with adhesion to the four described human GP2 isoforms. FimH variants bound similarly to porcine and human GP2: high-, low-, and no-binding variants were identical using porcine and human GP2 (**Figure 5A**). Thus, GP2 does not seem to be a host-specific selector for *Salmonella* serotypes.

We tested whether FimH variant binding was specific for GP2 and included two standard glycoproteins: horseradish peroxidase (HRP) and RNase B. In general, there was no difference between binding to GP2 and binding to other glycoproteins: if variants bound well to GP2, these variants also bound well to HRP and RNase B (**Figure 5A**). Finally, binding to glycoproteins was always mannose-dependent (**Figure 5B**) and glucose-independent (data not shown) which additionally indicates FimH-mannose residue interaction.

### Generation of GP2-Expressing Cell Lines

**FIGURE 6:**
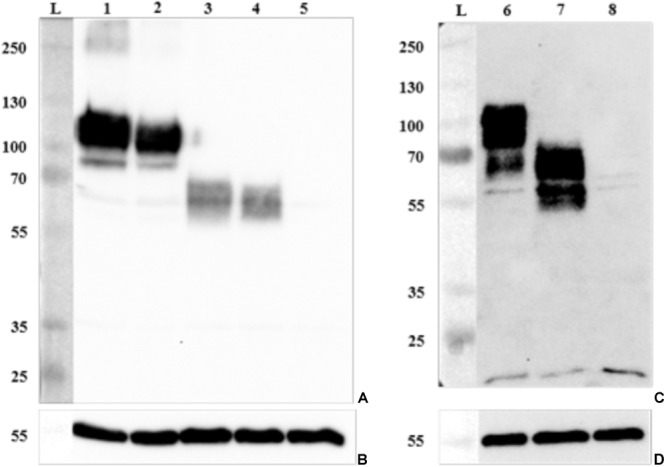
Detection of GP2 expression in epithelial cells by Western blotting. Proteins of cell lysates were separated by SDS–PAGE and GP2 and α tubulin (control) expression was confirmed with **(A)** an antibody against human GP2 isoform 1 (anti-GP2Ho#1 antibody) expressed in HEp-2 cells, **(B,D)** an antibody against α tubulin, and **(C)** an antibody against porcine GP2 isoform 2 (anti-GP2Su#2 antibody) expressed in IPEC-J2 cells. Lane L: protein ladder (kDa); lane 1: HEp-2 cells expressing human GP2 isoform 1 (HEp-2-GP2Ho#1); lane 2: HEp-2-GP2Ho#2; lane 3: HEp-2-GP2Ho#3; lane 4: HEp-2-GP2Ho#4; lane 5: HEp-2 cells transduced with an empty lentivirus (control, HEp-2-pLVX-Empty); lane 6: IPEC-J2-GP2Su#1; lane 7: IPEC-J2-GP2Su#2; lane 8: IPEC-J2-pLVX-Empty.

Both porcine GP2 isoforms with appropriate GPI anchor were expressed in porcine epithelial cells (IPEC-J2) and human GP2 isoforms 1–4 were similarly expressed in human epithelial cells (HEp-2). Optimal selection of all transduced cells was found using 1 μg/ml puromycin. GP2 expression was confirmed on the mRNA level by RT-PCR (data not shown) and on protein level by IIF (**Supplementary Figure 3**), Western blotting (**Figure 6**), and flow cytometry (**Supplementary Figure 4**). IIF and flow cytometry always indicated GP2 expression in more than 90% of cells. Additionally, flow cytometry validation showed that all cell lines expressed similar amounts of surface GP2 (**Supplementary Figure 4**). As negative control (empty lentivirus control), cell lines transduced with “empty” lentiviruses not-containing GP2 sequences did not express GP2 (**Supplementary Figure 4**).

### Cell Line Adhesion/Infection Assays

We finally explored the GP2 – FimH interaction by infection assays using the isogenic strains. For automated counting of bacteria during infection assays of a cell line, we adapted our automated fluorescence microscopy platform “VideoScan” for counting fluorescent bacteria stained by FISH (**Supplementary Figure 5**). This included detection and quantification of FISH-stained bacteria and enabled us to screen infectivity of all FimH variants to all cell lines expressing GP2 isoforms in a 96-well format including large amounts of repeats and negative controls.

**FIGURE 7:**
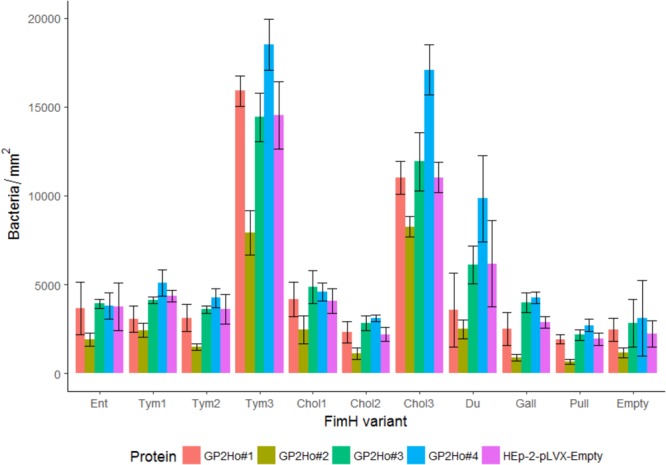
Isogenic *Salmonella* strains infect HEp-2 cells which express GP2. FimH isogenic strains of Tym5744Δ*fimH* were incubated with HEp-2 cells expressing human GP2 isoforms 1–4 (HEp-2-GP2Ho#1–4). For comparison HEp-2-pLVX-Empty cells not expressing GP2 were also infected. After infection, non-adherent bacteria were removed by washing. Cells and bacteria were fixed. Bacteria were stained by FISH. Bacteria (adherent + invasive) were counted by the fluorescence microscopy technology VideoScan and presented as bacteria per mm^2^. Ent: FimH variant of *S*. Enteritidis, Tym1-3: FimH variants of *S*. Typhimurium, Chol1-3: FimH variants of *S*. Choleraesuis, Du: FimH variant of *S*. Dublin, Gall: FimH variant of *S*. Gallinarum, Pull: FimH variant of *S*. Pullorum, Empty: FimH mutant without FimH complementation. The data are shown as median values and median absolute deviation (MAD) of three separate experiments in quadriplicate wells.

**FIGURE 8:**
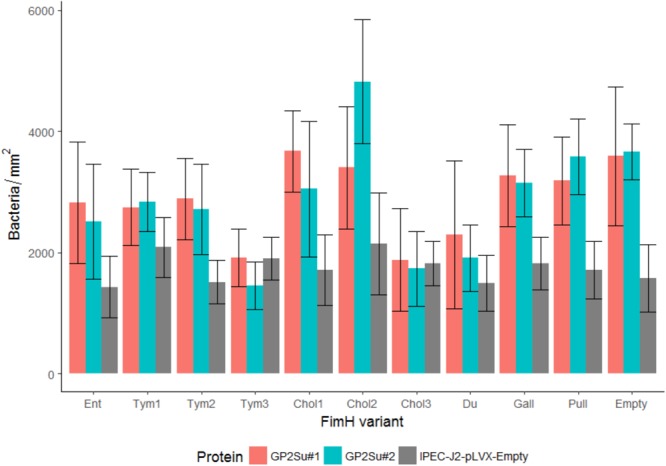
Isogenic *Salmonella* strains infect IPEC-J2 cells which express GP2. FimH isogenic strains of Tym5744Δ*fimH* were incubated with IPEC-J2 cells expressing porcine GP2 isoforms 1 and 2 (IPEC-J2-GP2Su#1 and 2). For comparison IPEC-J2-pLVX-Empty cells ( = empty lentivirus control) not expressing GP2 were also infected. After infection, non-adherent bacteria were removed by washing. Cells and bacteria were fixed. Bacteria were stained by FISH. Bacteria (adherent + invasive) were counted by the fluorescence microscopy technology VideoScan and presented as bacteria per mm^2^. Ent: FimH variant of *S*. Enteritidis, Tym1-3: FimH variants of *S*. Typhimurium, Chol1-3: FimH variants of *S*. Choleraesuis, Du: FimH variant of *S*. Dublin, Gall: FimH variant of *S*. Gallinarum, Pull: FimH variant of *S*. Pullorum, Empty: FimH mutant without FimH complementation. The data are shown as median values and median absolute deviation (MAD) of five separate experiments in quadriplicate wells.

Infection assays included – similar to the static adhesion assay – the *Salmonella* FimH variants as well as the FimH mutant which was transformed with the same vector like the FimH variants but without any *FimH* gene ( = empty plasmid control). In general, all isogenic *Salmonella* strains were able to infect host cells not expressing GP2 as well as host cells expressing GP2. There were differences in the two cell line models. Using HEp-2 cells (expressing and not expressing GP2) an infection pattern similar to the static GP2 adhesion assay was visible. Three high binding FimH variants (Tym3, Chol3, and Du) adhered better than others (*p* < 0.05), underlining the importance of the FimH sequence for cell line infection (**Figure 7**). Adding mannose into cultures infected by Tym3, Chol3, and Du resulted in a decrease in infection, suggesting that these strains are mannose sensitive. However, FimH variant Tym1 which bound well in the static adhesion assay did not infect cells better than the empty plasmid control. In contrast, using IPEC-J2 cells, there was not such obvious FimH variant-dependent infection. All FimH variants and the empty plasmid control rather similarly infected cells (**Figure 8**).

Another difference in the two cell lines was revealed by comparing cells not expressing and cells expressing GP2. The empty plasmid control (absence of FimH) infected all five HEp-2 cell lines in similar quantity, indicating that all cell lines were infected in a similar FimH-GP2-independent way (**Figure 7**). In contrast, in IPEC-J2 cells the empty plasmid control infected better the GP2 expressing than the GP2 not expressing cells (*p* < 0.05). This points to an alternative adhesion mechanism which is induced by knocking out the *FimH* gene and which also uses porcine GP2 in IPEC-J2 cells (**Figure 8**).

Finally, we analyzed whether GP2 expression results in higher infection by *Salmonella* FimH variants. In HEp-2 cells, there was no general trend that GP2 expression supports infection indicating that the surface of HEp-2 cells already presents sufficient glycoproteins other than GP2 as receptors. However, some differences were seen like FimH variants Tym3 and Chol3 significantly better infected HEp-2 cells expressing human GP2 isoform 4. But same Tym3 and Chol3 also significantly worse infected HEp-2 cells expressing human GP2 isoform 2 compared to HEp-2 wild type cells and HEp-2 cells expressing the two other isoforms (*p* < 0.05), though – already mentioned above – all isoforms were similarly expressed on the host cell surface (**Supplementary Figure 4**). This indicates a complex interplay of glycoproteins on cells and that different GP2 isoforms can affect infection by different FimH variants.

In IPEC-J2 cells, all FimH variants which did not bind to GP2 in the static adhesion assay (Ent, Tym2, Chol1, Chol2, Gal, Pul) bound better to GP2-expressing cells compared to the original cells, which was almost always significant (*p* < 0.05). In contrast, all FimH variants which well bound to GP2 in the static adhesion assay (Tym1, Tym3, Chol3, Du) did not infect GP2-expressing cells better compared to cells not expressing GP2. This points to an alternative adhesion mechanism which is induced by lacking a FimH-mediated adhesion process and which also uses porcine GP2 as receptor in IPEC-J2 cells (**Figure 8**).

## Discussion

Due to the high homology of GP2 with the Tamm-Horsefall protein (THP, uromodulin) various possible functions of THP were implicated to GP2. According to current findings, GP2 secreted into the intestinal lumen may serve as an immunomodulator and compound sterically hindering binding of bacteria to host epithelial cells (Yu and Lowe, 2009; Werner et al., 2012). The discovery of GP2 expression on the surface of intestinal M cells added a new possible function of GP2 as receptor for T1F-positive bacteria (Roggenbuck et al., 2009; Ohno and Hase, 2010). We proposed that various FimH variants could have different affinities to GP2 isoforms of pigs and humans (Kolenda, 2018).

FimH of *E. coli* interacts with the glycan part of GP2 and several studies showed that the requirement for any protein to be considered as a receptor for T1F is glycosylation (Leusch et al., 1991; Kukkonen et al., 1993; Yu and Lowe, 2009; Grzymajlo et al., 2013). Our deglycosylation experiments confirmed glycosylation of recombinant GP2 used in this study and that *Salmonella* FimH binds to the glycan part of GP2 similarly to *E. coli* FimH. Of note, there is only one protein (plasminogen) that *Salmonella* T1F bind in a non-mannose-sensitive manner (Kukkonen et al., 1998).

FimH variation can have a considerable impact on adhesion to receptors. Out of a collection of 128 *Salmonella* isolates including the clinically most important serotypes, we report two new FimH variants (Chol1 and Tym2) which were not reported so far in GenBank. Additionally, eight already described variants were confirmed. Most of *S*. Typhimurium isolates contained the Tym1 FimH variant. It has been demonstrated that Tym1 with Val at residue 245 can be more often found in human but Tym 3 with Ala more in animal isolates (Yue et al., 2015). Surprisingly, we identified five human isolates with Tym3 in this study, but found no porcine equivalents. It has to be mentioned that Chol1 was found in *S*. Choleraesuis var. Decatur and this strain was isolated from a reptile which might explain the different sequence since other isolates belonged to *S*. Choleraesuis *sensu stricto* isolated from pigs, wild boars, pork, or related products. In *S*. Dublin, we identified only one FimH variant which has the identical sequence of the only published FimH variant in this serotype, underlining a high conservation. Gall and Pull FimH variants found in our strain collection represent the most often found FimH sequences for these serotypes/biotypes (Kisiela et al., 2005, 2006).

We investigated the interaction between GP2 and FimH using a previously validated and published method, the static adhesion assay (Schierack et al., 2015). This method was used to compare the binding between FimH variants, expressed on cells with identical genetic background (isogenic model), to GP2 isoforms expressed in SF9 cells. We used SF9 cells to express the proteins as this allows for post synthetic modification of recombinantly expressed mammalian glycoproteins. In parallel, using anti-FimH antibodies we tested for FimH expression. FimH expression varied between FimH variants – though expressed in the isogenic background – and could not be improved by varying multiple passages. We have no plausible explanation or hypothesis for the variation in FimH expression levels between variants. However, similar variations have been reported before for *Salmonella* FimH (Kisiela et al., 2012). However, normalization per FimH expression did not change our observations and conclusions (**Supplementary Figure 2**). Non-binding of Gall and Pull to GP2 confirmed our experimental setting. This is in line with previous data identifying Gall and Pull non-binding to be mediated by a single amino acid substitution in the FimH sequence (Kisiela et al., 2005). Regarding the Chol FimH variants, we found one non-binding (Chol2), one low-binding (Chol1), and one high-binding (Chol3) phenotype. The presence of non-binding Chol2 and high-binding Chol3 was reported previously (Kisiela et al., 2012) and additionally added validity to our experimental setting. The presence of low-binding Chol1 was not reported in the literature so far. The only reported low-binding Chol1 isolate belonged to a different biotype of *S*. Choleraesuis (biotype Decatur) according to the White-Kauffmann-Le Minor scheme, and was isolated from a reptile (Issenhuth-Jeanjean et al., 2014). Isolates from the *S*. Choleraesuis biotype Decatur were reassigned in the publication of Achtman et al. (2012) as *S*. Decatur. Therefore, multilocus sequence typing or next generation sequencing of strains bearing Chol FimH variants could help to establish genetic relatedness between these strains, and explain whether isolates bearing a Chol1 variant should be considered as low binding FimH variant of *S*. Choleraesuis or *S*. Decatur. It has been proposed that T1F production in *S*. Choleraesuis is dependent on the presence of Gly at residue 63 (like Chol2), but our results showed that both Gly and Val at residue 63 resulted in fimbriated phenotypes (Lee and Yeh, 2016). The binding properties of Tym1, Du, and Ent in our study are in agreement with previous results (Grzymajlo et al., 2010). Tym2 and Tym3 represented low and high binding phenotypes reported previously. However, they were associated with different amino acid substitutions at different loci. This suggests that evolutionary adaption in *S*. Typhimurim selects for mutations leading to these two binding phenotypes (Boddicker et al., 2002).

We could globally define binding patterns of different FimH *Salmonella* variants to all glycoproteins, ranging from those that did not bind to very high binders. Each variant that could bind to one glycoprotein, either as a low-binder or as a high-binder, bound similarly to all other glycoproteins. This shows that the FimH sequence and the FimH expression determines the binding characteristics rather than the glycoprotein which in our case were recombinantly expressed GP2, native HRP from horseradish, and native bovine pancreatic RNase B.

In a next step, we studied the FimH-GP2 interaction in a more natural but also more sophisticated environment. We expressed GPI-anchored GP2 isoforms in cell lines and established a new FISH analysis software module for bacteria quantification. The conventional reference assay to determine the number of bacteria infecting cell lines is the colony-forming unit (CFU) assay by plating serial dilutions of bacteria suspensions on LB agar (Lee and Falkow, 1990). Other methods include staining of bacteria (GFP, antibody) and manual bacteria counting with fluorescence microscopy or enumeration with flow cytometry (Santos et al., 2013; Knodler et al., 2014). The aforementioned methods are not suitable for cell line infection studies using larger amounts of bacterial strains and cell lines. Our VideoScan technology (Rödiger et al., 2013) enabled screening of 10 FimH variants on six cell lines expressing GP2 and additional controls.

In general, there were significant differences between HEp-2 and IPEC-J2 cells. HEp-2 cells: *Salmonella* FimH variants infected HEp-2 cells in a similar manner to adhesion in the static adhesion assay. FimH variants Tym3, Chol3, and Du infected cells better than other variants. This underlines the importance of the FimH sequence for determining *Salmonella* infectivity as already proposed by Dwyer et al. (2011) and Kisiela et al. (2012). With few exceptions, FimH variants infected cells expressing GP2 as well as those not expressing GP2. This is in line with the observation of the static adhesion assay where FimH variants bound similarly to all GP2 isoforms and other glycoproteins. Furthermore, this shows that the HEp-2 cell surface is already saturated with glycoproteins other than GP2, and that *Salmonella* FimH can use these as alternative receptors. However, since intestinal GP2 expression levels can change (Roggenbuck et al., 2009), the glycoprotein or GP2 isoform pattern might also change and affect bacterial binding. In our study, expression of GP2 isoform 4 in HEp-2 cells resulted in increased *Salmonella* infection but expression of GP2 isoform 2 in HEp-2 cells resulted in decreased infection, though expression of both GP2 isoforms was similar as measured by flow cytometry. This indicates a sophisticated glycoprotein expression network in cells. Further work is necessary to study expression of GP2 isoforms in different intestinal parts and in different cell types as well as to compare healthy and diseased hosts to clarify roles of different GP2 isoforms in infection.

Regarding IPEC-J2 cells, we did not reveal a FimH variant-dependent infection. Likewise, in the static adhesion assay Tym3 bound more to glycoproteins than did Tym1 or Tym2. In contrast, in the cell infection assay Tym3 infected similarly to Tym1 or Tym2. This was seen for the cells expressing GP2 as well as for the cells not expressing GP2. The same was seen for the empty plasmid control lacking FimH which similarly infected IPEC-J2 cells compared to all FimH variants. Thus, an alternative adhesion/infection process presumably existed in IPEC-J2 cells and this alternative process (1) solely mediated infection or (2) overlayed the FimH-mediated adhesion process. That a more prominent adhesion mechanism can cover T1F adhesion was shown by Kleta et al. (2014). Adhesion of one *E. coli* strain to IPEC-J2 cells was prominently mediated by F1C fimbriae which covered adhesion mediated by T1F (Kleta et al., 2014).

GP2-expressing IPEC-J2 cells were infected better by *Salmonella* isogenic strains expressing no-binding and low-binding FimH variants or without FimH expression (the empty plasmid control), compared to the IPEC-J2 cells without GP2 expression. This was not the case for high-binding Tym1, Tym3, Chol3, and Du of the static adhesion assay. These variants similarly infected GP2-expressing and non-GP2-expressing cells. It seems that if a FimH variant was not able to recognize glycoproteins then this variant expressed an alternative adhesion process finally resulting in GP2 recognition. This supports rather the hypothesis, that an existing FimH-GP2 interaction was covered by an alternative adhesion mechanism instead of the full absence of a FimH-GP2 interaction. A mutual impact of one adhesin on the expression of another adhesin is a so-called “regulatory cross-talk between adhesions.” Such direct communication has been shown between the two adhesin gene clusters T1F and pyelonephritis-associated pili in uropathogenic *E. coli* (Xia et al., 2000) and for regulation of expression of flagellar, SPI1, and type 1 fimbrial genes in *Salmonella* (Saini et al., 2010). In other studies, an *E. coli* mutant that was incapable of expressing either T1F or P fimbriae compensated by synthesizing F1C fimbriae (Snyder et al., 2005) and *E. coli* and *Salmonella* regulators of S, P, and long polar fimbriae inhibited T1F expression (Holden et al., 2001). The alternative *Salmonella* adhesion process in our study and the alternative adhesin-specific binding to GP2 have to be defined in future studies.

One of the goals of this work was to demonstrate interactions between FimH and GP2, that could support a role of GP2 in host specificity. However, our data do not indicate such a role of any *Salmonella* FimH variant in host specificity, because there were no clear differences in binding to GP2 isoforms from different species in both assays. For example, Tym3 which is a FimH variant of host-unrestricted *S*. Typhimurium as well as Chol3 which is a FimH variant of host-restricted *S*. Choleraesuis preferentially infected human HEp-2 but not porcine IPEC-J2 cells. Thus, *Salmonella* serovars can use GP2 as receptor during pathogenesis, but it is not specific to the host with which they are associated. That there were both high and low GP2-binding FimH variants in host generalists (*S*. Enteritidis as well as *S*. Typhimurium) and that *Salmonella* isolates of both human and porcine origin did not contain any unique/host-related FimH variant underlines that GP2-FimH interaction is rather not a mechanism of host tropism.

## Conclusion

In pigs, there are two functional GP2 isoforms expressed which differ in their amino acid sequence to human GP2 isoforms by roughly 25%. We defined out of 128 *Salmonella* isolates two new and 8 already published FimH variants each of them only found in one serotype. FimH variants could be assigned to a high-, low-, or no-binding phenotype. In a static adhesion assay, FimH interactions with glycoproteins was FimH variant-dependent but not specific for GP2 isoforms or host source. In infection assays using one human and one porcine cell line model, there were fundamental differences. Using HEp-2 cells, infection was FimH variant-specific but with few exceptions independent of GP2 expression in general or independent of one human GP2 isoform. Using IPEC-J2 cells, FimH variant-dependent infections were not visible. In FimH variants which were not able to recognize glycoproteins, an alternative adhesion/infection process was triggered which resulted in enhanced infection of GP2-expressing cells. Thus, *Salmonella* can express other adhesins than FimH which specifically uses GP2 as host cell receptor. Finally, GP2 was not a receptor determining host-specificity of *Salmonella*. Both *Salmonella* specialists and generalists can bind similarly to GP2.

Future studies should test whether GP2 is a relevant receptor for FimH *in vivo* and in different hosts systems. Studies also have to focus on the spatial distribution of GP2 isoforms in the human and porcine intestine and their different expression under health and disease. Changes in a cell surface glycoprotein pattern including GP2 isoform pattern could increase the importance of GP2 in the ensemble of glycoproteins. Finally, other studies should focus on other bacteria species since a pathotype-specific binding of *E. coli* to GP2 was already determined (Schierack et al., 2015).

## Author Contributions

RK, SR, DR, and PS performed the conception and design. RK, MB, SR, DR, and PS performed the data analysis. RK, JS, LS, IS, AO, JW, JN, AB, and UG performed the data acquisition. RK, DR, and PS wrote the manuscript. All the authors gave their final approval of the version to be published.

## Conflict of Interest Statement

DR is a shareholder of GA Generic Assays GmbH and Medipan GmbH, both are diagnostic manufacturers. The remaining authors declare that the research was conducted in the absence of any commercial or financial relationships that could be construed as a potential conflict of interest.
