# Supplementary material for: Adhesion of Salmonella to Pancreatic Secretory Granule Membrane Major Glycoprotein GP2 of Human and Porcine Origin Depends on FimH Sequence Variation
Source: Front Microbiol. 2018 Aug 22;9:1905. doi: 10.3389/fmicb.2018.01905 (PMC6113376; doi:10.3389/fmicb.2018.01905)
Supplement: Supplementary file 1 [file Data_Sheet_1.DOCX]

Supplementary Material

**Adhesion of *Salmonella* to pancreatic secretory granule membrane major glycoprotein GP2 of human and porcine origin depends on FimH sequence variation**

**Rafał Kolenda^1,2^, Michał Burdukiewicz^3^, Juliane Schiebel^1^, Stefan Rödiger^1^, Lysann Sauer^1^, Istvan Szabo^4^, Aleksandra Orłowska^4^, Jörg Weinreich^1^, Jörg Nitschke^1^, Alexander Böhm^1^, Ulrike Gerber^1^, Dirk Roggenbuck^1,5^, Peter Schierack^1,*^**

Institute of Biotechnology, Faculty Environment and Natural Sciences, Brandenburg University of Technology Cottbus-Senftenberg, Senftenberg, Germany^1^

Department of Biochemistry, Pharmacology and Toxicology, Wrocław University of Environmental and Life Sciences, Poland^2^

Department of Genomics, Faculty of Biotechnology, University of Wrocław, Poland^3^

National *Salmonella* Reference Laboratory, Federal Institute for Risk Assessment (BfR), Berlin, Germany ^4^

GA Generic Assays GmbH, Dahlewitz, Germany^5^

***Correspondence:**

Peter Schierack
Institute of Biotechnology
Faculty Environment and Natural Sciences
Brandenburg University of Technology Cottbus-Senftenberg
Universitätsplatz 1, 01968 Senftenberg, Germany
Tel.: 03573 / 85-932, [Peter.schierack@b-tu.de](mailto:Peter.schierack@b-tu.de)

**1. Supplementary Figures and Tables**

**
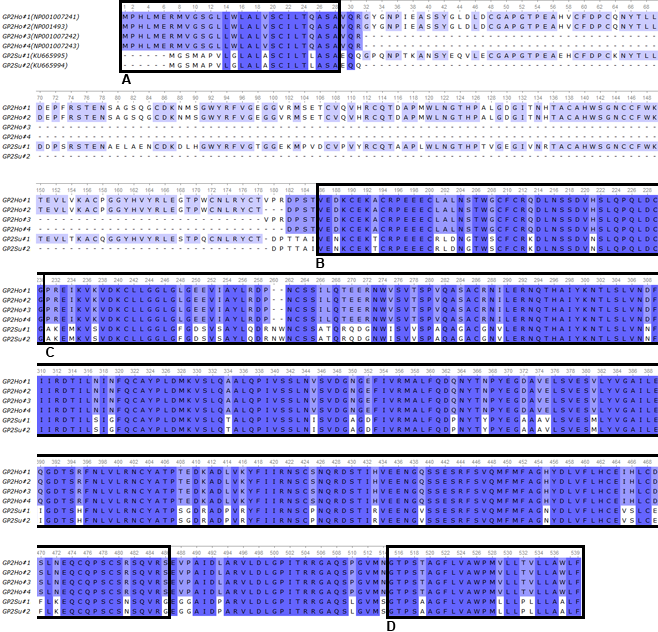
1.1 Supplementary Figures**

**Supplementary Figure 1.** Alignment of amino acid sequences of GP2 isoforms. Compared are full amino acid sequences of human GP2 isoforms 1 to 4 (GP2Ho#1 to 4) and porcine GP2 isoforms 1 and 2 (GP2Su#1 and 2). The sequence data are available from GenBank under the indicated accession numbers (NP001007241 (GP2Ho#1), NP001007242 (GP2Ho#3), NP001007243 (GP2Ho#4), P001493 (GP2Ho#2), KU665995 (GP2Su#1), KU665994 (GP2Su#2). A: N-terminal signal peptide, B: EGF-like domain, C: zona pellucida (ZP) domain, D: C-terminal glycosylphosphatidylinisotol (GPI)-anchor.

**
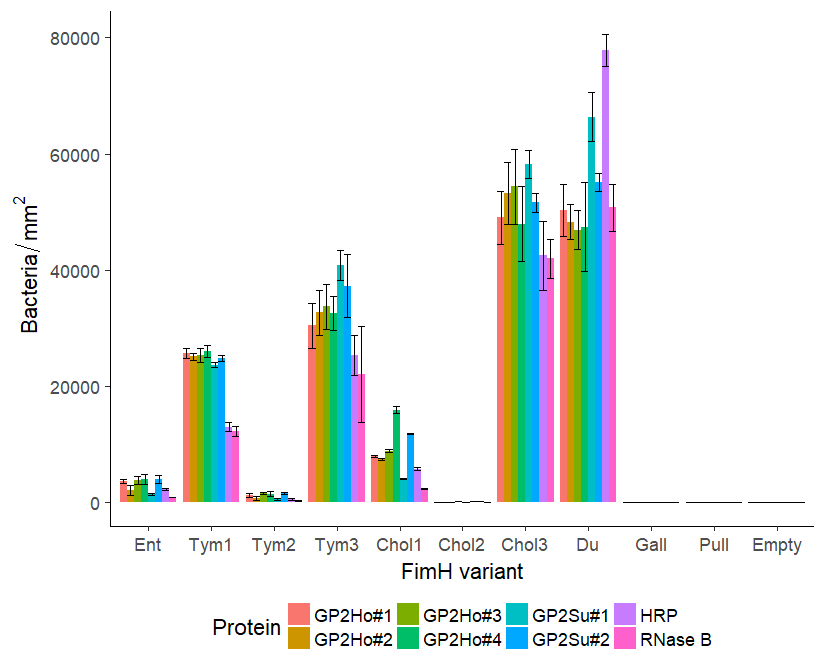
**

**Supplementary Figure 2. Binding of *Salmonella* to glycoproteins – normalization relative to FimH expression level.** FimH isogenic strains of Tym5744Δ*fimH* were incubated with various proteins including human GP2 isoforms 1-4 (GP2Ho#1-4), porcine GP2 isoforms 1-2 (GP2Su#1-2), horseradish peroxidase (HRP) and RNase B. All strains in the isogenic recombinant system expressed FimH, which was shown by staining of FimH (please see figure 4 of the main manuscript). However, FimH expression varied between FimH variants. Thus we normalized binding to glycoproteins relative to FimH expression. We chose Tym3 as reference point since this FimH variant was the most expressed. We calculated the factor between FimH expression of Tym3 and each other variant and recalculated FimH expression of all FimH variants. Thus, each FimH variant was virtually expressed at the same level. Using this factor, we calculated binding of each FimH variant to glycoproteins. Conclusively, all numbers except for Tym3 increased. However, this resulted in the same outcome for our interpretation like in Figure 4: Tym1, Tym3, Chol3 and Du were high binding phenotypes; Ent, Tym2 and Chol1 were low binding phenotypes; and Chol2, Gall and Pull were no-binding phenotypes. If one FimH variant bound well to one GP2 isoform then this variant bound also bound well to other isoforms of the host species. If one FimH variant bound well to human GP2 this variant also bound well to porcine GP2. Ent: FimH variant of *S*. Enteritidis, Tym1-3: FimH variants of *S*. Typhimurium, Chol1-3: FimH variants of *S*. Cholerasuis, Du: FimH variant of *S*. Dublin, Gall: FimH variant of *S*. Gallinarum, Pull: FimH variant of *S*. Pullorum, Empty: FimH mutant without FimH complementation. The data are shown as median values and median absolute deviation (MAD) of three separate experiments in triplicate wells.


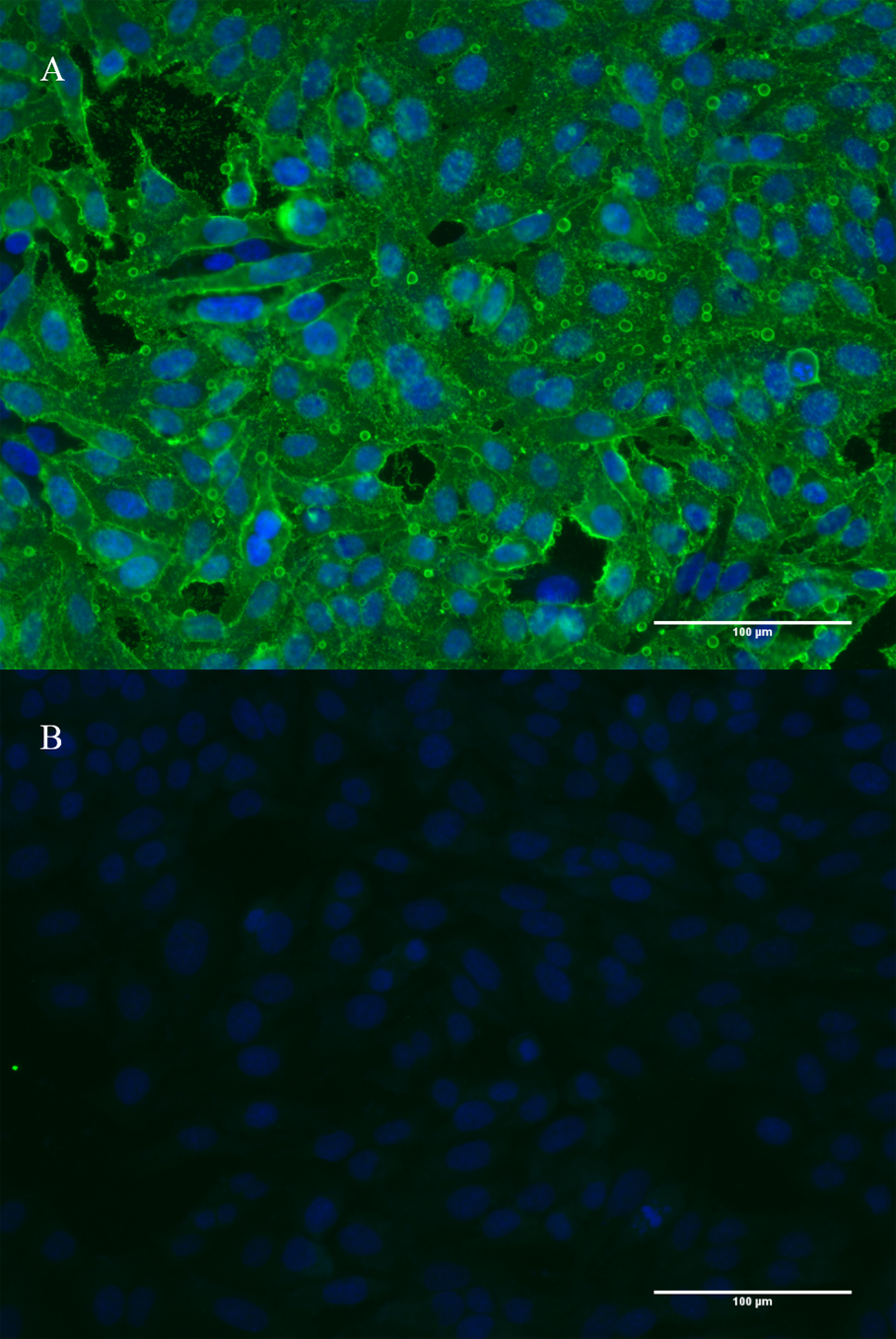


**Supplementary Figure 3.** Detection of GP2 expression in epithelial cells by indirect immunofluorescence. Shown is one example of A) HEp-2 cells expressing human GP2 isoform 2 (HEp-2-GP2Ho#2) and B) HEp-2 cells transduced with an empty vector (HEp-2-pLVX-Empty). GP2 was stained with anti-GP2 antibodies and a secondary antibody conjugated with FITC (green). Cell nuclei are stained with DAPI (blue).


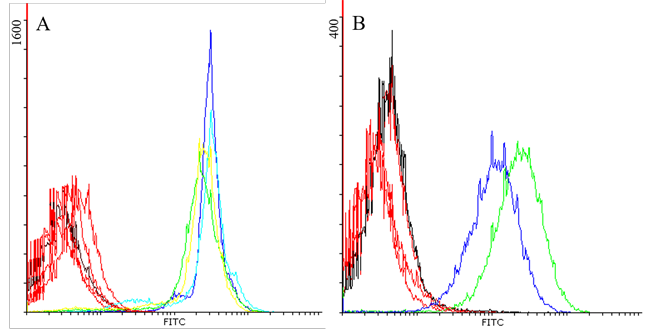


**Supplementary Figure 4. Detection of GP2 expression in epithelial cells by flow cytometry.** GP2-expressing cells were stained with antibodies and measured by flow cytometry. A) GP2 expressed in HEp-2 cells and stained with antibody against human GP2 isoform 1 (anti-GP2Ho#1 antibody); blue: HEp-2 cells expressing human GP2 isoform 1 (HEp-2-GP2Ho#1); cyan: HEp-2-GP2Ho#2; green: HEp-2-GP2Ho#3; yellow: HEp-2-GP2Ho#4; black: HEp-2 cells transduced with an empty vector; red: control, secondary antibody staining all HEp-2 cell lines. B) GP2 expressed in IPEC-J2 cells and stained with antibodies against porcine GP2 isoforms 1 and 2 (anti-GP2Su#1 and anti-GP2Su#2); green: IPEC-J2 cells expressing porcine GP2 isoform 1 (IPEC-J2-GP2Su#1); blue: IPEC-J2-GP2Su#2; black: IPEC-J2 cells transduced with an empty vector; red: control, secondary antibody staining all IPEC-J2 cell lines.


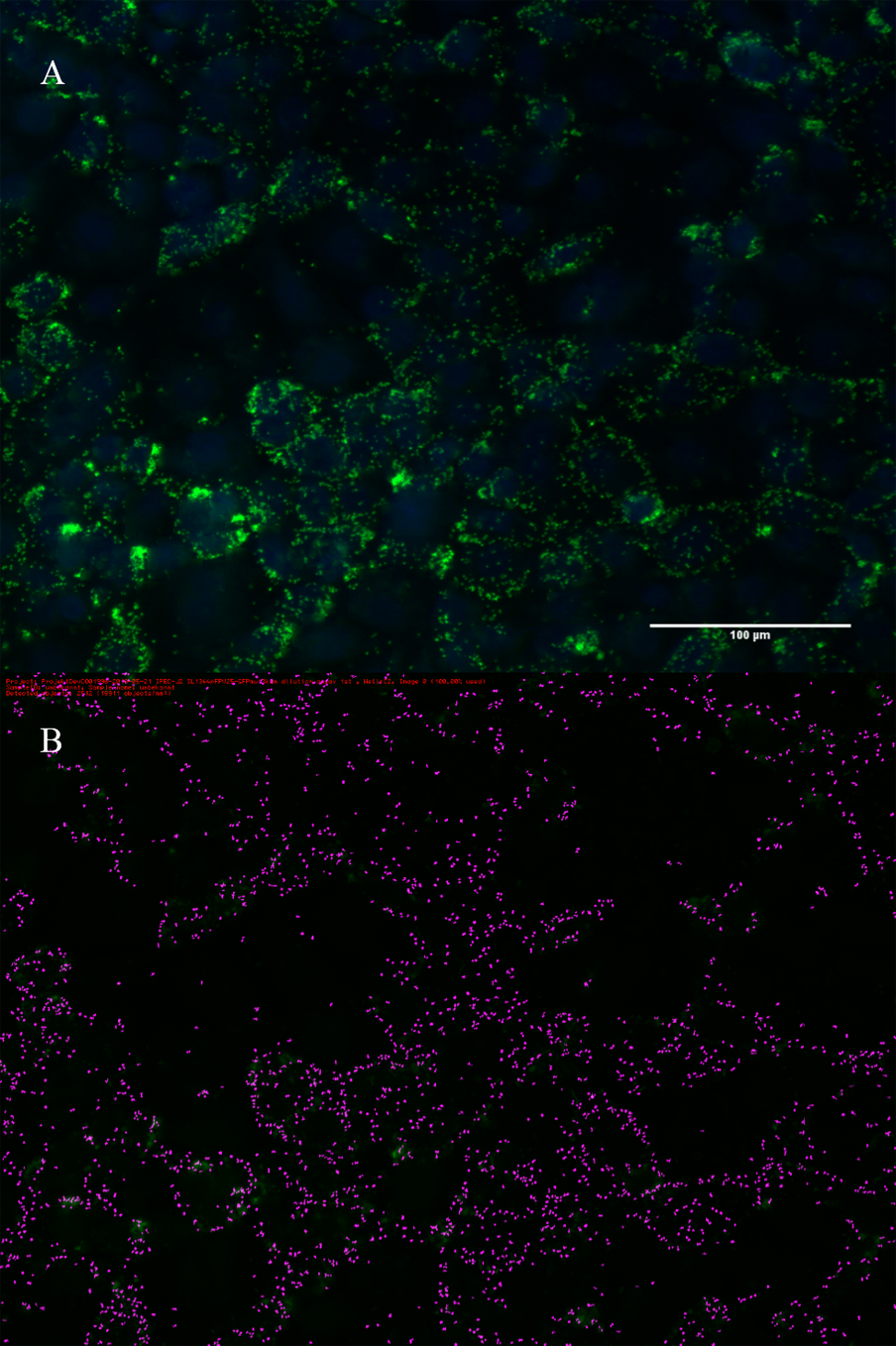


**Supplementary Figure 5. Quantification of *Salmonella* infection by the VideoScan technology.** Epithelial cells were infected with *Salmonella* isogenic strains in a 96-well format. *Salmonella* was stained by fluorescence in situ hybridization (FISH). Images were automatically taken by the VideoScan instrument; A) one exemplary image with stained *Salmonella* (Atto647, green) and cell nuclei (DAPI, blue); B) Image A processed with VideoScan, recognized and counted bacteria marked in magenta.

**1.2 Supplementary Tables**

**Supplementary Table 1.** **List of the strains.**

| Strains (abbreviations) | Description | Source |
| --- | --- | --- |
| *Escherichia coli* XL1Blue | *recA1 endA1 gyrA96 thi-1 hsdR17 supE44 relA1 lac [F´ proAB lacIq Z∆M15 Tn10 (Tetr )]* | Agilent Technologies |
| *Escherichia coli* DH10Bac | F– *mcr*A Δ(*mrr*-*hsd*RMS-*mcr*BC) φ80*lac*ZΔM15 ΔlacX74 *rec*A1 *end*A1 *ara*D139 Δ(*ara*, *leu*)7697 *gal*U *gal*K λ– *rps*L *nup*G/bMON14272/pMON7124 | Invitrogen |
| *Salmonella enterica* subsp*. enterica* serovar Typhimurium (Tym-human) | 20 strains, isolated from human diarrhea cases | Mydlak/Thorasch Diagnostic Laboratory, Cottbus |
| *Salmonella enterica* subsp*. enterica* serovar Typhimurium (Tym-pig) | 20 strains, isolated from porcine stool samples | BfR, Berlin |
| *Salmonella enterica* subsp*. enterica* serovar Enteritidis (Ent-human) | 20 strains, isolated from human diarrhea cases | Mydlak/Thorasch Diagnostic Laboratory, Cottbus |
| *Salmonella enterica* subsp*. enterica* serovar Enteritidis (Ent-chicken) | 14 strains, isolated from chicken stool samples | BfR, Berlin |
| *Salmonella enterica* subsp*. enterica* serovar Gallinarum (Gall) | 19 strains, isolated from chicken stool samples | BfR, Berlin |
| *Salmonella enterica* subsp*. enterica* serovar Dublin (Du) | 20 strains, isolated from bovine stool samples | BfR, Berlin |
| *Salmonella enterica* subsp*. enterica* serovar Choleraesuis (Chol) | 15 strains, isolated from various sources (porcine stool, wild boars, meat products, reptile) | BfR, Berlin |

**Supplementary Table 2. List of primers used in this study.**

| No. | Primer Name | Gene(-s)/ Target | Primer sequence (5’-3’) | Primer Length | T_m_ | Reference |
| --- | --- | --- | --- | --- | --- | --- |
| 1. | O1545 GP2SuFor | GP2 | ATGGGAAGCATGGCTCCC | 18 | 62.0 | This study |
| 2. | O1546 GP2SuRev | GP2 | TCAGAACAGCGCAGCCAG | 18 | 63.0 | This study |
| 3. | pJET1.2 forward sequencing primer | pJET1.2 | CGACTCACTATAGGGAGAGCGGC | 23 | 60.5 | Kit manual |
| 4. | pJET1.2 reverse sequencing primer | pJET1.2 | AAGAACATCGATTTTCCATGGCAG | 24 | 62.0 | Kit manual |
| 5. | O1628 GP2SuFor | GP2 | GGGGACAAGTTTGTACAAAAAAGCAGGCTATACCATGGGAAGCATGGCTCCC | 52 | 62.0 | This study |
| 6. | O1739 GP2SuRevGate3 | GP2 | GGGGACCACTTTGTACAAGAAAGCTGGGTCTTAATGGTGATGGTGATGGTGTCCACTTCCACTTCCCATGACGCCAAGAGACTGG | 85 | 61.0 | This study |
| 7. | pUC/M13 Forward | Bacmid | CCCAGTCACGACGTTGTAAAACG | 23 | 64.5 | Kit manual |
| 8. | pUC/M13 Reverse | Bacmid | AGCGGATAACAATTTCACACAGG | 23 | 61.0 | Kit manual |
| 9. | O1532 fimH_Sal_For | *fimH* | ATCCAGTGGGGAGAGGG | 17 | 60.0 | This study |
| 10. | O1533 fimH_Sal_Rev | *fimH* | GAGTTGGCCTGACTCAGC | 18 | 60.5 | This study |
| 11. | O1901 fimHdelfwd | *fimH* | ATGAAAATATACTCAGCGCTATTGCTGGCGGGGACCGCGCTCTTTTTCACTGTGTAGGCTGGAGCTGCTTC | 71 | 62.5 | Zeiner et al., 2012 |
| 12. | O1902 fimHdelrev | *fimH* | TTAATCATAATCGACTCGTAGATAGCCGCGCGCAGTAAACGGCCCTTCCGCATATGAATATCCTCCTTAG | 70 | 52.3 | Zeiner et al., 2012 |
| 16. | O1919 FimHpACYCfor | *fimH* | ACATGGATCCTTGACAATTAATCATCGGCTCGTATAATGTGTGGAGGAGGACAGCTATGAAAATATACTCAGCGCTATTG | 80 | 58.5 | This study |
| 17. | O1920 FimHpACYCrev | *fimH* | ACATGGATCCTTAATCATAATCGACTCGTAGATAG | 35 | 59.0 | This study |
| 18. | O1461 GP2HoEcoRIFor | GP2 | GTCACGAATTCATGCCTCACCTTATGGAAAGG | 32 | 60.0 | This study |
| 19. | O1462 GP2HoBamHIRev | GP2 | CGTGGATCCTCAGAACAGCCAAGCCAGG | 28 | 62.5 | This study |
| 20. | O1590 GP2SuFor | GP2 | GTCACGAATTCATGGGAAGCATGGCTCCC | 29 | 62.0 | This study |
| 21. | O1591 GP2SuRevBam | GP2 | CGTGGATCCTCAGAACAGCGCAGCCAG | 27 | 63.0 | This study |
| 22. | O1573 GP2HoqPCR2for | GP2 | ATCAACGTGATTCCACCATCC | 21 | 61.0 | This study |
| 23. | O1574 GP2HoqPCR2rev | GP2 | TTGAGCAAGAAGGCTGGC | 18 | 61.0 | This study |
| 24. | O2229 GP2SuForqPCR2 | GP2 | AACAGCTCGGATGTCAACAG | 20 | 61.0 | This study |
| 25. | O2230 GP2SuRevqPCR2 | GP2 | AGATCCAGTTCCCATCCTGTC | 21 | 61.5 | This study |
| 26. | O876 RPLP0_fw | RPLP0 | AAATGTTTCATTGTGGGAGC | 20 | 57.0 | George et al.,2016 |
| 27. | O877 RPLP0_rv | RPLP0 | ATATGAGGCAGCAGTTTCTC | 20 | 58.0 | George et al.,2016 |
